# Reconstitution of the Jasmonate Signaling Pathway in Plant Protoplasts

**DOI:** 10.3390/cells8121532

**Published:** 2019-11-28

**Authors:** Ning Li, Joachim F. Uhrig, Corinna Thurow, Li-Jun Huang, Christiane Gatz

**Affiliations:** 1 State Key Laboratory of Cultivation and Protection for Non-Wood Forest Trees, Ministry of Education, Central South University of Forestry and Technology, Hunan 410004, China; nli@csuft.edu.cn; 2 College of Forestry, Central South University of Forestry and Technology, Hunan 410004, China; 3 Albrecht-von-Haller-Institute for Plant Sciences, Georg-August-University Göttingen, 37077 Göttingen, Germany; juhrig@uni-goettingen.de (J.F.U.); cthurow@uni-goettingen.de (C.T.)

**Keywords:** jasmonic acid, signaling pathway, protoplasts, transient gene expression

## Abstract

The phytohormone jasmonic acid (JA) plays an important role in various plant developmental processes and environmental adaptations. The JA signaling pathway has been well-elucidated in the reference plant *Arabidopsis thaliana*. It starts with the perception of the active JA derivative, jasmonoyl-isoleucine (JA-Ile), by the F-box protein COI1 which is part of the E3-ligase SCF^COI1^. Binding of JA-Ile enables the interaction between COI1 and JAZ repressor proteins. Subsequent degradation of JAZ proteins leads to the activation of transcription factors like e.g., MYC2. Here we demonstrate that the pathway can be reconstituted in transiently transformed protoplasts. Analysis of the stability of a JAZ1-fLuc fusion protein as a function of COI1 transiently expressed in *coi1* protoplasts allows structure function analysis of both JAZs and COI1. Using this system, we found that conserved cysteines in COI1 influence steady state COI1 protein levels. Using a luciferase reporter gene under the control of the *JAZ1* promoter enable to address those features of JAZ1 that are required for MYC2 repression. Interestingly, the conserved TIFY-motif previously described to interact with NINJA to recruit the corepressor TOPLESS is not necessary for repression. This result is in favor of the alternative repression mode that proposes a direct competition between repressive JAZs and promotive MEDIATOR25 at MYC2. Finally, using protoplasts from the *aos* *coi1* double mutant, which is deficient in JA synthesis and perception, we provide a system that has the potential to study the activity of different COI1 variants in the presence of different ligands.

## 1. Introduction

Plants are constantly exposed to a wide variety of environmental stresses that negatively affect their growth and yield. Specific signaling networks serve to activate specific genes that serve to combat adverse conditions and to coordinate plant growth accordingly [1,2,3]. Understanding the molecular mechanisms underlying those signaling pathways is key to designing plants with an improved stress tolerance and growth performance. The fatty acid-derived plant hormone jasmonic acid (JA) regulates a number of plant developmental and environmental responses [4,5,6,7]. JA is produced by the oxylipin biosynthesis pathway [8,9]. Linolenic acid is converted into JA via sequential reactions conducted by various biosynthesis enzymes, including lipoxygenase, allene oxide synthase (AOS), allene oxide cyclase, and oxophytodienoate reductase [4,10]. Finally, JA is conjugated to isoleucine through the activity of a jasmonoyl-L-amino acid synthetase to form the major bioactive JA derivative, jasmonoyl-isoleucine (JA-Ile) [11,12].

In the past few decades, combinations of genetic, biochemical, and protein structural analyses have identified the core JA signaling components in the model plant *Arabidopsis thaliana* [13,14,15]. JA-Ile triggers the interaction between the F-box protein CORONATINE INSENSITIVE1 (COI1) and the JASMONATE-ZIM DOMAIN (JAZ) transcriptional repressor proteins, which function together as JA coreceptors [16,17,18]. COI1 recruits *Arabidopsis* Skp1-like1 (ASK1) and Cullin1 (CUL1) to form the Skp1/Cullin1/F-box protein COI1 (SCF^COI1^) complex that serves as an E3 ubiquitin ligase to target the JAZ repressors to ubiquitination and subsequent degradation via the *26S* proteasome [14,19]. In the absence of JA-Ile, JAZs repress the MYC2 transcription factor [20,21,22]. The *Arabidopsis* JAZ protein family has 12 members. All JAZ proteins contain two conserved domains: the ZIM-domain containing the TIFY-motif required for complex formation with Novel Interactor of JAZ (NINJA) and heteromeric interactions between JAZ proteins and the JAZ domain that contains the Jas-motif mediating COI1 and MYC2 interactions [22,23,24]. NINJA recruits TOPLESS (TPL) and TPL-related (TPR) transcriptional corepressors and is required for repression in roots [25]. The COI1-mediated degradation of JAZ repressors upon JA sensing leads to the dissociation of the transcriptional repressor complex, which, in turn, releases MYC2 transcriptional activity [7,26].

Studies of plant signaling pathways are labor-intensive and time-consuming [27,28]. The protoplast transient gene expression system offers invaluable opportunities for obtaining additional information including subcellular localization of signaling compounds, interaction studies, and analysis of protein mutants [29,30]. In this study, we aimed to reconstitute the JA signaling pathway using the protoplast transient gene expression system. By using known features of the key signaling proteins COI1 and JAZ1 we proved that the pathway works as described for differentiated plant cells. Moreover, we addressed the function of two conserved cysteines in COI1 and the role of the conserved TIFY-motif in JAZ1. Thus, we show that this pathway reconstitution in protoplasts represents a rapid and reliable means for structure function analysis of key players of the pathway.

## 2. Materials and Methods

### 2.1. Plant Materials and Growth Conditions

All plants used in this study are in the *Arabidopsis thaliana* accession Columbia (Col-0) background. *Arabidopsis aos* (*dde2-2*; [31]) and *coi1* (SALK_035548; [32]) mutant seeds were obtained from Prof. Beat Keller (University of Zurich, Zurich, Switzerland) and Prof. Ingo Heilmann (Martin-Luther-University of Halle-Wittenberg, Germany), respectively. The *aos* mutant was crossed with the *coi1* mutant to generate the *aos coi1* double mutant [33]. For experiments including *coi1* mutation, seeds were sown on Murashige and Skoog (Duchefa, Haarlem, Netherlands) agar plates containing 50 µM methyl jasmonate (Sigma-Aldrich, Darmstadt, Germany) to select homozygous plants [34]. Plants were grown in soil in growth cabinets (Percival Scientific, Germany) at 22 °C, 60% relative humidity, 80–100 µmol photons m^−2^ s^−1^, 12-h-light/12-h-dark photoperiod, and 60% relative humidity, until being used for the experiments.

### 2.2. Construction of Recombinant Plasmids

For transient gene expression in protoplasts, the recombinant reporter and effector plasmids were generated following standard molecular biology protocols and GATEWAY cloning technology (Invitrogen, Carlsbad, CA, USA).

We developed two types of reporter plasmids: the protein-fusion reporter and the promoter-driven reporter. To obtain the GATEWAY (GW) destination vector *UBQ10pro*:HA-GW-fLuc, the *Hind*III and *Xma*I fragments containing the firefly *luciferase* sequence from the pBGWL7.0 vector [35] were inserted into the same restriction sites of the *UBQ10pro*:HA-GW vector [36]. The *JAZ1* (AT1G19180) coding sequence was amplified with GATEWAY adapter primers JAZ1-gw-d1 and JAZ1ohnestop-gw-r1 (primer sequences are shown in Table 1). The PCR product flanked by the *attB* sequence was cloned into the intermediate vector pDONR207 (Invitrogen, Carlsbad, CA, USA), according to the manufacturer′s instructions. The resulting entry clone pDONR207-JAZ1ohnestop was recombined with the destination vector *UBQ10pro*:HA-GW-fLuc to produce the protein-fusion reporter vector *UBQ10pro*:HA-JAZ1-fLuc. Generation of the mutant JAZ1 (mJAZ1) derivative was achieved by PCR using pDONR207-JAZ1ohnestop as a template with primer pairs SeqL1/mJAZ1-r1 and mJAZ1-d1/SeqL2, resulting in two fragments that served as a template for overlapping PCR with primers SeqL1 and SeqL2. The mJAZ1 fragment was recombined into *UBQ10pro*:HA-GW-fLuc to produce the *UBQ10pro*:HA-mJAZ1-fLuc vector.

To construct the promoter-driven reporter vector, the 1267 bp promoter region of the *JAZ1* gene was amplified using *Arabidopsis* genomic DNA as a template with primers JAZ1pro-gw-d1 and JAZ1pro-gw-r1. The fragment was recombined into pDONR207. The resulting entry clone pDONR207-JAZ1pro was recombined with the destination vector pBGWL7.0, yielding the promoter-driven reporter vector *JAZ1pro*:fLuc with the *JAZ1* promoter sequence upstream of the firefly *luciferase* reporter gene.

To construct effector vectors, the destination vector *UBQ10pro*:HA-GW was used. Full-length *COI1* and *JAZ1* coding sequences, including a stop codon, were amplified with primer pairs COI1-gw-d1/COI1mitstop-gw-r1 and JAZ1-gw-d1/JAZ1mitstop-gw-r1, respectively, and recombined into the pDONR207 vector to produce pDONR207-COI1 and pDONR207-JAZ1. pDONR207-COI1, -JAZ1, and -MYC2 [33] were then recombined with the destination vector *UBQ10pro*:HA-GW to produce the effector vectors *UBQ10pro*:HA-COI1, -JAZ1, and -MYC2. Three PCR reactions were used to obtain the mJAZ1 coding sequence, including a stop codon, one reaction used the pDONR207-JAZ1 as template with primers SeqL1/mJAZ1-r1. A second PCR reaction used the pDONR207-JAZ1 as template with primers mJAZ1-d1/SeqL2. The PCR products from these two reactions containing complementary sequences were used as templates in a third PCR reaction with primers SeqL1/SeqL2. The product was recombined with the destination vector *UBQ10pro*:HA-GW to produce the effector vector *UBQ10pro*:HA-mJAZ1. The generation of mCOI1 derivative was achieved by the same overlapping PCR approach. A first reaction used the pDONR207-COI1 as template with primers SeqL1/mCOI1-r1. A second PCR reaction used the pDONR207-COI1 as template with primers mCOI1-r1/SeqL2. The PCR products from these two reactions were used as templates in a third PCR reaction with primers SeqL1/SeqL2. The product was recombined with the destination vector *UBQ10pro*:HA-GW to produce the effector vector *UBQ10pro*:HA-mCOI1. The generation of COI1SS, COI1SC, and COI1CS mutant derivatives were achieved by a similar overlapping PCR approach. A first reaction used the pDONR207-COI1 as template with primers seqL1/COI1CC-r1. A second PCR reaction used the pDONR207-COI1 as template with primer pairs COI1SS-d1/SeqL2, COI1SC-d1/SeqL2, and COI1CS-d1/SeqL2, respectively. The PCR products from these two reactions containing complementary sequences were used as templates in a third PCR reaction with primers SeqL1/SeqL2. The products were recombined with the destination vector *UBQ10pro*:HA-GW to produce the effector vectors *UBQ10pro*:HA-COI1SS, *UBQ10pro*:HA-COI1SC, and *UBQ10pro*:HA-COI1CS, respectively. All constructs were sequenced by Microsynth Seqlab (Hannover, Germany).

### 2.3. Protoplasts Preparation and Transfection

Protoplast assays were carried out as described previously by Yoo et al. [30] and Li et al. [37], using four-week-old soil-grown *Arabidopsis thaliana* wild-type and mutant plants. All chemicals used in this study were obtained from Sigma-Aldrich (Darmstadt, Germany), unless otherwise stated.

The lower surface of *Arabidopsis* leaves was lightly scratched with a razor blade and placed in a Petri dish (SARSTEDT, Germany) containing 10 mL enzyme solution (1.5% cellulase R10, 0.4% macerozyme R10 (SERVA, Heidelberg, Germany), 400 mM mannitol, 10 mM CaCl_2_, 20 mM KCl, 20 mM MES-KOH, pH 5.7). After incubation overnight under a 12/12 light condition the digested solution was filtered through a 75 µm mesh, and the protoplasts were centrifuged (780 rpm, 2 min, soft start and stop). The pellet was washed twice with 10 mL W5 solution (154 mM NaCl, 125 mM CaCl_2_, 5 mM KCl, 2 mM MES-KOH, pH 5.7) and afterwards, the protoplasts were resuspended in W5 solution and incubated on ice before transfection. For polyethylene glycol (PEG)-mediated transfection of the protoplasts, the W5 solution covering the protoplasts was removed carefully and the pellet was resuspended in MMG solution (400 mM mannitol, 15 mM MgCl_2_, 4 mM MES-KOH pH 5.7). Protoplasts in MMG solution (200 µL per transfection) were transferred into a 2 mL safe-lock round-bottom microtube (SARSTEDT, Germany) containing 220 µL 40% (*w*/*v*) PEG solution and 20 µL plasmid DNA mix (5.0 µg effector plasmids, 5.0 µg reporter plasmids, and 1.0 µg reference plasmids). The solution was gently mixed and incubated at room temperature for 30 min. Then, 800 µL W5 buffer was added and gently mixed by inverting the tube. After centrifugation at 780 rpm for 2 min, the supernatant was removed and the pellet was resuspended in 300 µL WI solution (500 mM mannitol, 20 mM KCl, 4 mM MES-KOH, pH 5.7), mixed gently, and incubated overnight under a 12/12 light condition. For chemical treatments, 5 µM JA-Ile (kindly provided by Prof. Mats Hamberg, Karolinska Institutet, Sweden) or coronatine (COR) was directly added to the WI solution. Four replicates were prepared for each transfection and all experiments were independently repeated at least three times.

### 2.4. Dual Luciferase Report Assay

Luciferase assays were performed according to Zander et al. [38]. Briefly, luciferase activities were measured using the Centro XS3 LB 960 microplate luminometer (Berthold Technologies, Germany) with the Dual-Luciferase Reporter Assay kit (Promega, Mannheim, Germany). After removing the WI solution, protoplasts were flash-frozen in liquid nitrogen. The frozen protoplasts were dissolved in 20 µL Passive Lysis Buffer and placed on ice. Afterwards, 3 µL of each lysate was transferred into a single well of a 348-multiwell plate (SARSTEDT, Germany), and each well was measured as follows: 30 s waiting time, injection of 15 µL LARII, 5 s waiting time, measurement of fLuc activity for 5 s, injection of 15 µL Stop&Glo, 5 s waiting time, and measurement of rLuc activity for 5 s.

### 2.5. Western Blot Analysis

The expression of hemagglutinin (HA)-tagged proteins in protoplasts was checked by Western blot analysis. After measuring luciferase activities, the rest of the lysate of four replicates was combined, treated with 6x protein extraction buffer (12% SDS, 60% glycerol, 0.06% bromophenol blue, 600 mM DTT, 375 mM Tris-HCl, pH 6.8), and heated at 60 °C for 10 min. Equal amounts of protein extracts (15 µL) were separated on 8% SDS polyacrylamide gel and subsequently transferred to polyvinylidene difluoride membranes (Carl Roth, Germany). Tagged proteins were detected with an anti-HA antibody (Santa Cruz Biotechnology, USA) and visualized with the SuperSignal^TM^ West Femto (Thermo Fisher Scientific, Germany) Western blot kit.

## 3. Results and Discussion

### 3.1. COI1-Mediated JAZ1 Protein Degradation in Arabidopsis Protoplast Cells

**Figure 1:**
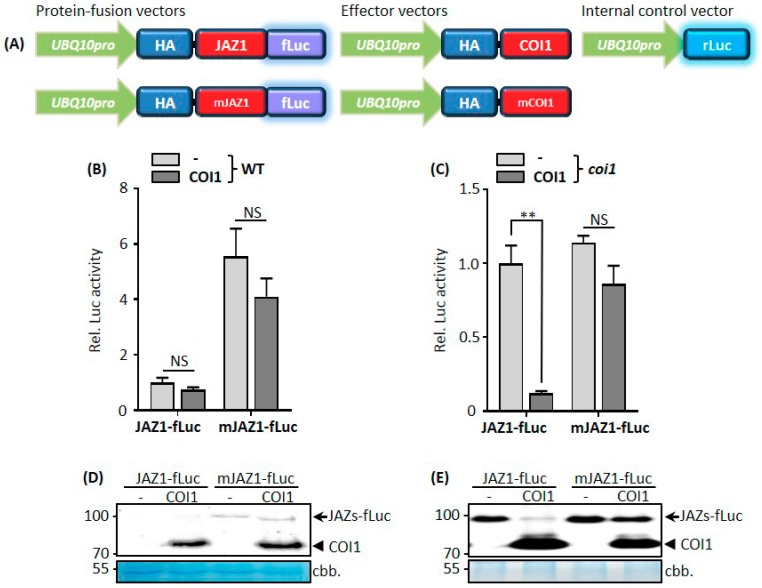
COI1-mediated JAZ1 protein degradation in *Arabidopsis* protoplast cells. (**A**) Scheme of the protein-fusion reporter, effector, and internal control vectors used for transient transfections. (**B**,**C**) Luciferase activities yielded by JAZ1-fLuc and mJAZ1-fLuc fusion proteins in the presence or absence of cotransfected COI1 in wild-type (WT) (**B**) and *coi1* (**C**) protoplasts. mJAZ1 contains R205A and R206A mutations. *Arabidopsis* leaf protoplasts prepared from WT or *coi1* mutant plants were cotransfected with protein-fusion reporter plasmids encoding JAZ1-fLuc or mJAZ1-fLuc under the control of the *UBQ10* promoter and effector plasmid encoding COI1 under the control of the *UBQ10* promoter. Firefly luciferase (fLuc) activities were normalized to *Renilla* luciferase (rLuc) activities. Reporter activities of JAZ1-fLuc without an effector vector were set to one. Values represent means (±SE) of four independently transformed batches of protoplasts. NS indicates not significant, ** *p* < 0.01, based on an unpaired, two-tailed Student′s *t*-test. (**D**,**E**) Western blot analysis was employed to detect levels of reporter and effector proteins. Four replicates from each transformation containing the same combination of plasmids as in (**B**,**C**) were combined for Western blot analysis using an anti- hemagglutinin (HA) antibody. Arrows denote JAZ1-fLuc or mJAZ1-fLuc fusion proteins, and the arrowheads denote COI1 proteins. ccb. denotes Coomassie Blue staining.

In order to investigate the degradation of JAZ proteins in protoplasts, we first developed a protein-fusion reporter construct (Figure 1A). To this aim, the *Arabidopsis UBIQUTIN-10* (*UBQ10*) gene promoter was placed upstream of the HA-tag fused in-frame to the GATEWAY cassette, followed by the firefly *luciferase* gene (*fLuc*). The GATEWAY cassette was replaced by the *JAZ1* coding gene to generate the final JAZ1 reporter construct *UBQ10pro*:HA-JAZ1-fLuc. In order to study the influence of COI1 on JAZ1-fLuc protein stability, we generated the COI1 effector construct *UBQ10pro*:HA-COI1. For the internal control construct used for normalization [36], the *Arabidopsis UBQ10* promoter was placed upstream of the *Renilla luciferase* gene (*rLuc*). Since the *UBQ10* promoter gives moderate, stable, and prolonged expression [39], we adopted it instead of the prevalent CaMV *35S* promoter for transient gene expression in *Arabidopsis* protoplasts.

The fLuc reporter activity provides a specific, sensitive, and quantifiable means to reflect the JAZ1-fLuc fusion protein abundances. We transiently expressed the JAZ1-fLuc reporter and COI1 effector in *Arabidopsis* protoplasts. As shown in Figure 1B, in *Arabidopsis* Col-0 wild-type (WT) protoplasts, the JAZ1-fLuc luciferase activity did not decrease after the addition of the COI1 effector plasmid, although its expression was confirmed by Western blot analysis (Figure 1D). Obviously, the endogenous COI1 activity in protoplasts is sufficient for degradation of additional amounts of JAZ substrates that were introduced. Next, we tested whether we could generate a stabilized protein based on previous Yeast-Two-Hybrid analysis that had shown that R205 and R206 of the Jas-motif play a critical role in mediating COI1–JAZ1 and COI1-JAZ9 interactions in the presence of the JA-Ile mimic coronatine (COR) [40]. Mutating R205 and R206 of JAZ1 conferred JA-insensitivity when the mutant protein was ectopically expressed in transgenic plants indicating that the missing COI1-interaction leads to stabilization of the JAZ protein and thus to permanent repression of the JA response. Our protoplast system directly proved these conclusions, since the JAZ1-fLuc protein was stabilized by exchanging the positively charged amino acid residues R205 and R206 within the COI1-interacting Jas-motif into alanine residues (Figure 1B).

Our data were confirmed by Western blot analysis, that were mainly designed to detect HA-tagged effector proteins. Whereas JAZ1-fLuc proteins were undetectable, the mJAZ1-fLuc proteins were detected both without and with additional expression of COI1 (Figure 1D). The comparison between the Western blot and the luciferase assay documents the higher sensitivity of the luciferase assay which still detects residual amounts of JAZ1-fLuc.

We took advantage of the *coi1* mutant to avoid JAZ1-fLuc degradation by endogenous COI1 in the protoplast system. In *coi1* protoplasts, the JAZ1-fLuc luciferase activity was comparable to that of the non-degradable mJAZ1-fLuc (Figure 1C). By the addition of COI1, the JAZ1-fLuc but not the mJAZ1-fLuc activity was reduced (Figure 1C). Western blot analysis confirmed equal expression of COI1 in the different samples and JAZ1-fLuc protein accumulation and degradation in the absence and presence of COI1, respectively (Figure 1E). The data obtained from the *coi1* mutant imply that synthetic rates of JAZ1-fLuc and mJAZ1-fLuc proteins were equal in protoplast cells. However, JAZ1-fLuc proteins synthesized in WT protoplasts were degraded by the protoplast endogenous COI1.

### 3.2. COI1-Mediated JAZ1 Protein Degradation in Protoplasts Requires Jasmonic Acid

**Figure 2:**
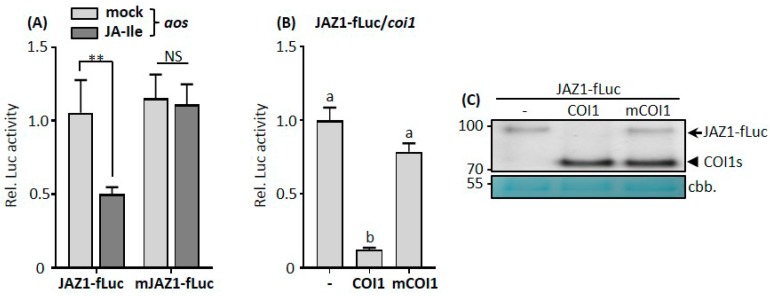
COI1-mediated JAZ1 protein degradation in protoplast cells requires jasmonic acid (JA). (**A**,**B**) Luciferase activities yielded by JAZ1-fLuc and mJAZ1-fLuc fusion proteins in the presence or absence of either JA-Ile (**A**) or a wild-type or mutated COI1 protein (**B**); mCOI1 has mutations in amino acids contacting JA-Ile; mJAZ1 contains mutations interfering with the interaction with COI1. *Arabidopsis* leaf protoplasts prepared from *aos* or *coi1* mutant plants were cotransfected with protein-fusion reporter plasmids encoding JAZ1-fLuc or mJAZ1-fLuc and effector plasmids encoding COI1 or mCOI1 under the control of the *UBQ10* promoter. For chemical treatment, the protoplasts in (A) were incubated without (mock) or with 5 μM JA-Ile after transfections. Firefly luciferase (fLuc) activities were normalized to *Renilla* luciferase (rLuc) activities. Reporter activities of JAZ1-fLuc without JA-Ile or an effector vector were set to one. Values represent means (±SE) of four independently transformed batches of protoplasts. For statistical analysis, NS and ** in (**A**) indicate not significant and *p* < 0.01, respectively, based on an unpaired two-tailed Student′s *t*-test; different letters in (**B**) denote significant differences between the respective effector plasmid combinations, based on one-way ANOVA with Tukey′s post hoc test, *p* < 0.01. (**C**) Western blot analysis was used to determine the expression levels of reporter and effector proteins. Four replicates from each transformation containing the same combination of plasmids as in (**B**) were combined for Western blot analysis using an anti-HA antibody after dual luciferase assays. The arrow denotes JAZ1-fLuc fusion proteins, and the arrowhead denotes COI1 or COI1 mutant variant (mCOI1) proteins.

In *Arabidopsis*, the endogenous JA-Ile molecules mediate the interaction between COI1 and JAZs. In order to determine whether the COI1-elicited JAZ1-fLuc proteolysis in protoplasts depends on JA, we performed a JA-Ile complementation assay in protoplasts prepared from *aos* plants. These plants are defective in the *AOS* gene encoding one of the key enzymes of the JA biosynthesis pathway and cannot synthesize the JA precursor oxophytodienoic acid [31,41]. In *aos* mutant protoplasts, JAZ1-fLuc and mJAZ1-fLuc represented the same Luc activities, suggesting that there was no degradation of JAZ1-fLuc proteins (Figure 2A). The incubation of *aos* protoplasts with exogenous JA-Ile led to a significant reduction of JAZ1-fLuc, but not mJAZ1-fLuc activity (Figure 2A). This result indicates the efficiency and specificity of JA-Ile in triggering JAZ1 degradation in protoplast cells.

Structural remodeling and biochemical data revealed that the bona fide JA-Ile receptor of *Arabidopsis* is a complex containing both COI1 and JAZ proteins [18]. The protein tertiary structure of COI1 contains a hormone-binding pocket assembled by leucine-rich repeats (LRRs) that binds the bioactive hormone JA-Ile with a high specificity and affinity. To test whether COI1 requires JA to facilitate degradation of JAZ proteins in the protoplast system, we generated a COI1 mutant variant (mCOI1), in which critical residues of the 13th LRR-motif (YMA_384_VYV) involved in JA-Ile binding were exchanged against the motif SVLYFC found in the structurally related auxin receptor TIR1 [17,42,43,44]. Due to compromised complex formation with JAZ proteins, mCOI1 was unable to degrade JAZ1-fLuc proteins in *coi1* protoplasts (Figure 2B). Western blot analysis showed that COI1 and mCOI1 proteins were expressed at a similar level (Figure 2C). JAZ1-fLuc proteins diminished only in the presence of WT COI1 (Figure 2C). These results indicate that COI1-mediated JAZ1 degradation in protoplasts requires JA perception and the amount of JA accumulating in protoplasts is sufficient to trigger such a process. Therefore, by transiently expressing the core JA receptor components in the JA biosynthesis or perception mutant protoplasts, we were able to establish a rapid and specific assay to monitor the JA-dependent and COI1-faciliated degradation of JAZ1 proteins, which is an early process of the JA signaling pathway.

**Figure 3:**
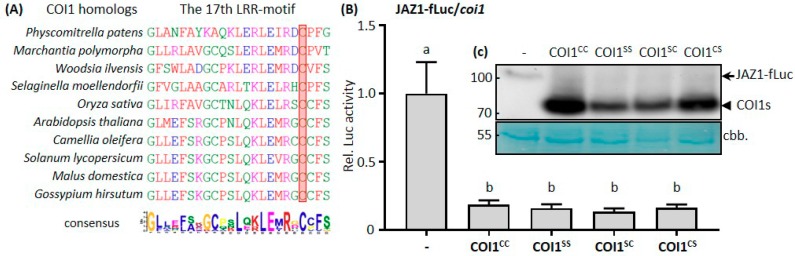
A conserved cysteine residue is important for COI1 stability, but not for COI1 function. (**A**) Sequence alignments of the 17th leucine-rich repeats (LRR)-motif of COI1 homologs from different plants. The conserved cysteine residue is highlighted in a red box. Plant species and accession numbers of COI1: *Physcomitrella patens* (XP_024401394.1); *Marchantia polymorpha* (BAS06563.1); *Selaginella moellendorfii* (Sm163526); *Oryza sativa* (Os01g0853400); *Arabidopsis thaliana* (NP_565919.1); *Solanum lycopersicum* (NP_001234464.1); *Malus domestica* (XP_008392915.2); *Gossypium hirsutum* (XP_016746378.1). The sequence of *Woodsia ilvensis* COI1 was extracted from the Phytozome database (http://www.phytozome.net) and the sequence of *Camellia oleifera* COI1 was found in our unpublished RNA-seq data. (**B**) The cysteine mutations show no effect on COI1 function. *Arabidopsis* leaf protoplasts prepared from *coi1* mutant plants were cotransfected with the protein-fusion reporter plasmid encoding *JAZ1* fused to firefly *luciferase* (JAZ1-fLuc) under the control of the *UBQ10* promoter and effector plasmids encoding derivatives of COI1 under the control of the *UBQ10* promoter. The two conserved cysteine residues of COI1 were singly or doubly replaced by serine residues. Firefly luciferase (fLuc) activities were normalized to *Renilla* luciferase (rLuc) activities. Reporter activities in the presence of an empty effector vector were set to one. Values represent means (±SE) of four independently transformed batches of protoplasts. Different letters denote significant differences, based on one-way ANOVA with Tukey′s post hoc test, *p* < 0.01. (**C**) Western blot analysis was used to assess expression levels of different COI1 proteins. Four replicates from each transformation containing the same combination of plasmids as in (**B**) were combined for Western blot analysis using an anti-HA antibody after dual luciferase assays. The arrow denotes JAZ1-fLuc fusion proteins, and the arrowhead denotes COI1 proteins.

Having established that the degradation of JAZ1-fLuc fusion protein can be used to study COI1 activity in *coi1* protoplasts, we addressed the functional significance of a cysteine residue close to the JA-Ile contacting amino acid R496 in the 17th LRR-motif that caught our attention due to its strong conservation from the lower land plant *Marchantia polymorpha* to all known sequences of higher plants (Figure 3A). In higher plants, this cysteine is followed by three amino acids leading to a CCFS motif, which resembles the active site (CCL/MS) of land-plant specific glutaredoxins [45]. Since cysteines are subject to redox modifications, we were interested whether mutating these residues into serines (C498S; C499S) would change the in vivo activity of COI1. We found that, in our protoplast system, single or double mutations of these conserved cysteines had no obvious impact in mediating JAZ1 degradation (Figure 3B). However, through Western blot analysis, we noticed that the mutation of C498 caused a reduction of COI1 protein accumulation (Figure 3C). Although we cannot exclude that the mRNA levels are affected or that the protein synthesis rate is altered, we consider it more likely that the mutated protein is less stable. Since the C/S mutation mimics the reduced state, one might speculate that oxidative modifications (e.g., glutathionylation, nitrosylation, or internal disulfide bridges) might stabilize the protein, while reducing conditions destabilize it. Whether this putative redox modulation of COI1 stability has any functional significance, remains to be shown. One possible scenario is that the phytohormone salicylic acid (SA), which lowers the redox potential of the cell [46,47], antagonizes the JA pathway by lowering COI1 amounts through this putative redox switch. Analysis of COI1 protein levels in SA treated samples would yield preliminary evidence to support the hypothesis.

### 3.3. COI1 Releases JAZ1-Repressed MYC2 Transcriptional Activity

After JA perception and the subsequent removal of JAZ repressors, the master transcription factor MYC2 is released to modulate JA-responsive gene expression [48,49]. MYC2 contains a typical DNA binding domain of the basic-helix-loop-helix motif that binds to the G-box *cis*-acting sequence (5′-CACGTG-3′) or its variants found in MYC2 target promoters [48]. To initiate transcription, MYC2 recruits the Mediator complex by physical association with the MED25 subunit [50,51]. MYC2 orchestrates the expression of early JA response genes that are involved in the regulation of specific branches of the JA signaling pathway. *JAZs* are early JA response genes and encompass G-box *cis*-elements in their promoter regions. Recent studies have demonstrated that MYC2 directly binds to the promoter of at least three *JAZ* genes, including *JAZ1* [21,52,53].

**Figure 4:**
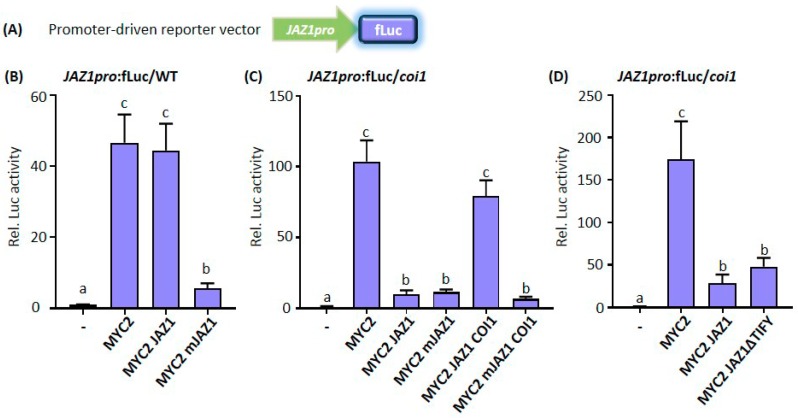
COI1 releases JAZ1-mediated repression of MYC2 transcriptional activity in protoplasts. (**A**) Scheme of the promoter-driven reporter vector. The JA-responsive *JAZ1* promoter sequence was fused to the firefly *luciferase* (fLuc) gene. (**B**) The JA-insensitive JAZ1 protein (mJAZ1) represses the MYC2 transcriptional activity in WT protoplasts. (**C**) The JAZ1-repressed MYC2 transcriptional activity is alleviated by COI1 in *coi1* protoplasts. (**D**) The conserved TIFY-motif is dispensable for JAZ1-repressive function. (**B**–**D**) *Arabidopsis* leaf protoplasts prepared from Col-0 or *coi1* mutant plants were cotransfected with the promoter-driven reporter plasmid encoding firefly *luciferase* under the control of the *JAZ1* promoter and effector plasmids encoding MYC2, JAZ1, mJAZ1, JAZ1ΔTIFY, or COI1 under the control of the *UBQ10* promoter. Firefly luciferase (fLuc) activities were normalized to *Renilla* luciferase (rLuc) activities. Reporter activities in the presence of an empty effector vector were set to one. Values represent means (±SE) of four independently transformed batches of protoplasts. Different letters denote significant differences between the respective effector plasmid combinations, based on one-way ANOVA with Tukey′s post hoc test, *p* < 0.01.

In order to investigate JA-induced transcriptional changes, the downstream process of JA signaling cascade, we constructed a promoter-driven reporter vector by fusing the *JAZ1* promoter to the fLuc reporter (*JAZ1pro*:fLuc; Figure 4A). In WT protoplasts, MYC2 strongly activated *JAZ1* promoter activity, and this activity was repressed by the non-degradable mJAZ1 but not by the WT JAZ1 (Figure 4B). This was consistent with data shown in Figure 1, which demonstrated that WT JAZ1 proteins were indeed degraded in WT protoplasts. Therefore, we performed the experiment in *coi1* mutant protoplasts. As shown in Figure 4C, MYC2-activated *JAZ1* promoter activity was stringently intercepted by both JAZ1 and mJAZ1 in *coi1* protoplasts. However, JAZ1- but not mJAZ1-mediated repression was alleviated by the addition of COI1 (Figure 4C). These data indicate the consistency and robustness of the protoplast system.

The TIFY-motif of JAZ proteins is indispensable for mediating the JAZ-NINJA interaction [22], which in turn is necessary to recruit the corepressor TPL and/or TPR proteins. However, NINJA is only required in roots for suppression of the JA response [25] and an alternative repression mode, which is based on the competitive inhibition of the MYC3 interaction with the MED25 subunit, has been put forward [54]. To test the repression mode in our protoplast system, we mutated the TIFY-motif in JAZ1. JAZ1 proteins without the TIFY-motif (JAZ1ΔTIFY) still repressed MYC2 transcriptional activity like the WT JAZ1 (Figure 4D). To our knowledge, this is the first time that the functional significance of the TIFY-motif for the repressive capacity of JAZ has been addressed and underlines the usefulness of the protoplast system described here.

### 3.4. JAZ1 Promoter Activity is Activated by an Exogenous JA Mimic and COI1 in Protoplasts

So far, we have established an experimental system, by which the ligand-dependent COI1-mediated JAZ1 degradation and the JAZ1-repressed MYC2 activity was analyzed by using either *coi1* or *aos* mutant protoplasts. In an attempt to rebuild a full JA signaling pathway in one protoplast assay, we used the previously described *aos coi1* double mutant plants [33], in which both JA biosynthesis and perception are deficient. As a reporter, we used the *JAZ1pro*:fluc construct that is inactive without any effectors due to weak expression of MYC2, which is repressed by stabilized JAZ proteins in this genetic background.

**Figure 5:**
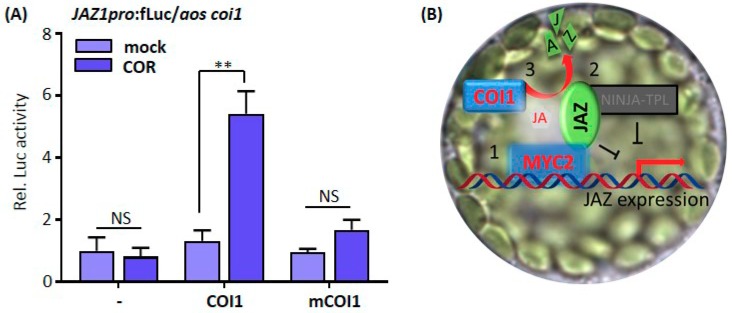
Reconstruction of the JA signal pathway in protoplast cells. (**A**) Activation of *JAZ1* promoter activity requires both exogenous JA mimic coronatine (COR) and COI1 in *aos coi1* protoplasts. *Arabidopsis* leaf protoplasts prepared from *aos coi1* double mutant plants were cotransfected with the promoter-driven reporter plasmid encoding firefly *luciferase* under the control of the *JAZ1* promoter and effector plasmids encoding derivatives of COI1 under the control of the *UBQ10* promoter. The transfected protoplasts were incubated without (mock) or with 5 μM COR after transfection. Firefly luciferase (fLuc) activities were normalized to *Renilla* luciferase (rLuc) activities. Reporter activities in the presence of an empty effector vector under a mock condition were set to one. Values represent means (±SE) of four independently transformed batches of protoplasts. NS indicates not significant, ** *p* < 0.01, based on an unpaired two-tailed Student′s *t*-test. (**B**) Diagrammatic representation of the major JA signal transduction events dissected in protoplast cells in this study.

We found that in the presence of both COI1 and COR, the *JAZ1* promoter activity was particularly increased in *aos coi1* protoplasts (Figure 5A). This reflected that endogenous MYC2 activity was released after the removal of JAZ repressors by COR-activated COI1. Again, the mCOI1 effector, that cannot form the COI1-COR-JAZ complex, was included as a negative control. The mCOI1 did not increase *JAZ1* promoter activity (Figure 5A). These data indicate that COR prompts COI1 to degrade JAZ repressors and relieves MYC2 activity in protoplasts, exactly as what we had known through genetic and biochemical analyses with intact *Arabidopsis* plants. Therefore, a full signaling cascade from early JA perception to downstream gene expression was successfully reconstituted in plant protoplasts (Figure 5B).

In summary, we reconstituted a JA signaling pathway, from JA perception and cover signal transduction to transcriptional regulation in protoplasts, which not only confirmed previous findings, but also facilitated the analysis of yet unknown JA signaling details. Previously, a transient expression assay in protoplasts and cell-free systems greatly facilitated identification of the conserved degron domain of Aux/IAA repressors that paved the way for the final defining of the auxin coreceptor complexes [55,56,57,58]. Fujii et al. reported an elegant reconstitution assay, with core abscisic acid (ABA) signaling components being sufficient to activate ABA-responsive gene expression in protoplasts [59]. The endogenous ABA signaling pathway seems inactive in WT protoplasts. Only recently, Ruschhaupt et al. reported the heterologous rebuilding of the *Arabidopsis* ABA signaling pathway in yeast [60].

## 4. Conclusions

In this study, by using gene knockout protoplasts, we were able to reconstitute the JA signaling pathway which provides an efficient, flexible, and versatile means of structure function analysis of signaling components. Moreover, the functionality of respective components from evolutionary distinct species can be addressed in the future. One limitation of the in vitro reconstitution in protoplasts, however, is that some results have to be validated in intact tissues and plants. The functional significance of altered COI1 stability after mutation of critical cysteines can only be addressed by stable complementation of the *coi1* mutant, after which the JA response can be studied in a more complex experimental set up including simultaneous treatment with salicylic acid or pathogen infections. Finally, this system allowed us to document that JAZ proteins can repress transcription in the absence of the conserved TIFY-motif.

## Figures and Tables

**Table 1:** A list of primer sequences used in this study.

Primer Symbol	Primer Sequence (5′-3′)
JAZ1-gw-d1	GGGGACAAGTTTGTACAAAAAAGCAGGCTCCATGTCGAGTTCTATGGAATG
JAZ1ohnestop-gw-r1	GGGGACCACTTTGTACAAGAAAGCTGGGTGATATTTCAGCTGCTAAACCG
JAZ1mitstop-gw-r1	GGGGACCACTTTGTACAAGAAAGCTGGGTGATCATATTTCAGCTGCTAAACCG
mJAZ1-d1	ACTTCCTATTGCTGCAGCAGCTTCAC
mJAZ1-r1	GTGAAGTGAAGCTGCTGCAGCAATAG
JAZ1ΔTIFY-d1	GTCTCAAACTGCACCATTGGCCGGGCAAGTGATTG
JAZ1ΔTIFY-r1	CAATCACTTGCCCGGCCAATGGTGCAGTTTGAGAC
COI1-gw-d1	GGGACAAGTTTGTACAAAAAAGCAGGCTCCATGGAGGATCCTGATATCAAGAGGTGT
COI1mitstop-gw-r1	GGGGACCACTTTGTACAAGAAAGCTGGGTCTCATATTGGCTCCTTCAGGACTC
mCOI1-d1	AGCTAGAATCGGTTCTCTACTTCTGCTCAGATATAACTAACGAATCTCTTGAAAG
mCOI1-r1	CTGAGCAGAAGTAGAGAACCGATTCTAGCTCCTGGCAGCCCTGAG
COI1SC-d1	GCTAGAGATGAGAGGTTCTTGCTTCAGTGAGCGAGCAAT
COI1CS-d1	GCTAGAGATGAGAGGTTGTTCCTTCAGTGAGCGAGCAAT
COI1SS-d1	GCTAGAGATGAGAGGTTCTTCCTTCAGTGAGCGAGCAAT
COI1CC-r1	AACCTCTCATCTCTAGCTTCTG
JAZ1pro-gw-d1	GGGGACAAGTTTGTACAAAAAAGCAGGCTCCCGCATAACAACAAAAACGTGG
JAZ1pro-gw-r1	GGGGACCACTTTGTACAAGAAAGCTGGGTGACATCTTTAACAATTAAAACTTTCAAAC
SeqL1	TCGCGTTAACGCTAGCATGGATCTC
SeqL2	GTAACATCAGAGATTTTGAGACAC
attB1-ad	GGGGACAAGTTTGTACAAAAAAGCAGGCT
attB2-ad	GGGGACCACTTTGTACAAGAAAGCTGGGT
